# Treatment of infected Achilles tendinitis and overlying soft tissue defect using an anterolateral thigh free flap in an elderly patient: A case report

**DOI:** 10.1097/MD.0000000000011995

**Published:** 2018-08-21

**Authors:** Young-Keun Lee, Malrey Lee

**Affiliations:** a Department of Orthopedic Surgery, Research Institute of Clinical Medicine of Chonbuk National University, Biomedical Research Institute of Chonbuk National University Hospital; b The Research Center for Advanced Image and Information Technology, School of Electronics and Information Engineering, Chonbuk National University, Jeonju, Chonbuk, Republic of Korea.

**Keywords:** Achilles tendon, anterolateral thigh perforator flap, reconstructive surgical procedures, wound infection

## Abstract

**Introduction:**

Infected segmental loss of the Achilles tendon with overlying soft tissue and skin defect remains a more complex reconstructive challenge. Here, we present a functional reconstruction of infected Achilles tendinitis with combined soft tissue defects using a free composite anterolateral thigh (ALT) flap with vascularized fascia lata in an elderly patient.

**Case presentation:**

A 71-year-old male patient was transferred to our department due to soft tissue defect of the left lower leg and infected Achilles tendinitis. The patient underwent incision and drainage of both lower legs with necrotizing fasciitis in another hospital 2 months ago. Physical examination revealed a 12 × 5 cm wound with exposed Achilles tendon over the posteromedial aspect of lower one-third of the leg. His wound culture grew methicillin-resistant *Staphylococcus aureus* (MRSA). All infected necrotic Achilles tendon with proximal muscle tissue was excised. The patient underwent successful Achilles tendon reconstruction and soft tissue coverage procedure with a 14 × 7 cm ALT flap with the fascia lata. At the 12-month follow-up, the patient resumed full daily activities, was able to squat, showed a range of motion at the ankle in the 15° dorsiflexion and 45° plantar flexion, and the American Orthopaedic Foot and Ankle Society (AOFAS) score was 94.

**Conclusion:**

A free ALT composite flap with the vascularized fascia lata, was successfully used for the treatment of infected Achilles tendinitis with overlying soft tissue defect even in an elderly patient. Furthermore, it provided satisfactory functional and cosmetic outcomes. Hence, the use of free ALT composite flap is highly recommended in such patients.

## 1 Introduction

Infected segmental loss of the Achilles tendon with overlying soft tissue and skin defect remains a more complex reconstructive challenge owing to the challenges associated with control of local infection, stable coverage, and restoration of active plantar flexion. Numerous reconstructive methods have been presented for the coverage of wound at the posterior heel.^[1]^ The one-stage reconstruction with composite-free tissue transplantation offers the advantages including radial healing, high resistance to infection, low morbidity, cost-effectiveness, decreased scarring and adhesion, and improved tendon gliding and excursion.^[2–6]^ Lee et al^[7]^ were the first to use a free composite anterolateral thigh (ALT) flap with vascularized fascia lata flap for the reconstruction of complex defects in 1999. The fascia lata receives sufficient blood supply via the prefascial and subfascial vascular plexuses when attached to the ALT flap. These attributes make the composite ALT flap a good alternative to other free composite flaps for combined reconstruction of Achilles tendon and soft tissue.^[4]^

In this report, we present our results involving functional reconstruction of infected Achilles tendinitis with combined soft tissue defects using a free composite ALT flap with vascularized fascia lata in an elderly patient.

## 2 Consent

The patient signed informed consent for publication of this case report and any accompanying images. The ethical approval of this study was waived by the ethics committee of Chonbuk National University Hospital because this study was a case report and fewer than 3 patients were involved.

## 3 Case report

A 71-year-old male patient was transferred to our department due to soft tissue defect in the left lower leg and infected Achilles tendinitis. The patient underwent incision and drainage of both lower legs with necrotizing fasciitis, at another hospital two months ago. Continuous wound care was performed; however, the left leg open Achilles tendinitis and soft tissue defects were not resolved.

**Figure 1:**
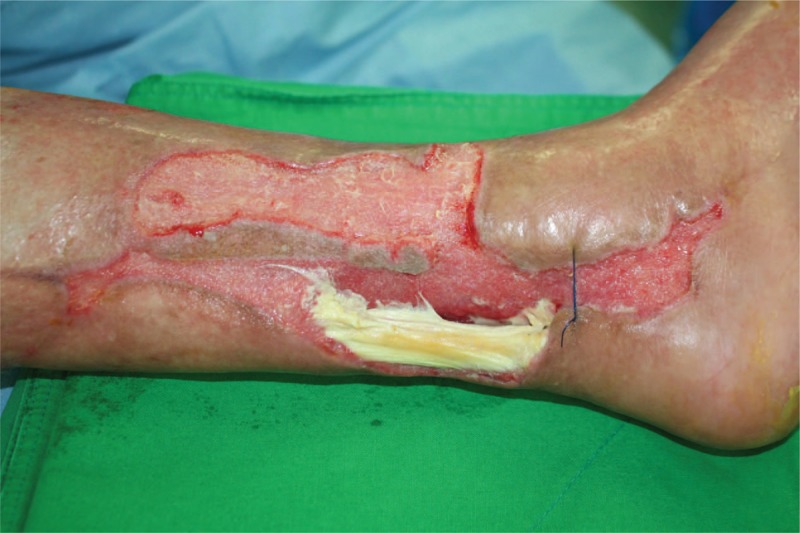
Intraoperative photograph showing the left Achilles tendon and soft tissue defects (12 × 5 cm) on the posteromedial aspect of lower one-third of leg.

Physical examination revealed a 12 × 5 cm wound with exposed Achilles tendon over the posteromedial aspect of lower one-third of the leg (Fig. 1). His wound culture grew methicillin-resistant *Staphylococcus aureus* (MRSA).

**Figure 2:**
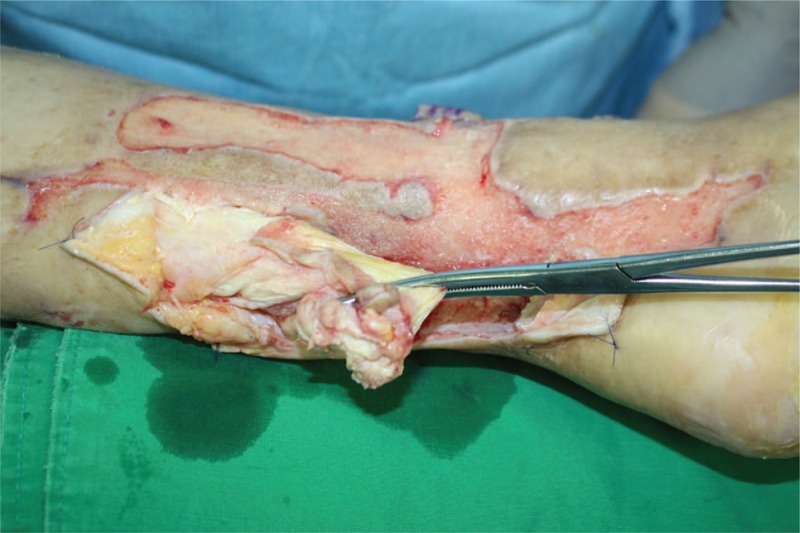
Intraoperative photograph showing removal of infected Achilles tendon.

**Figure 3:**
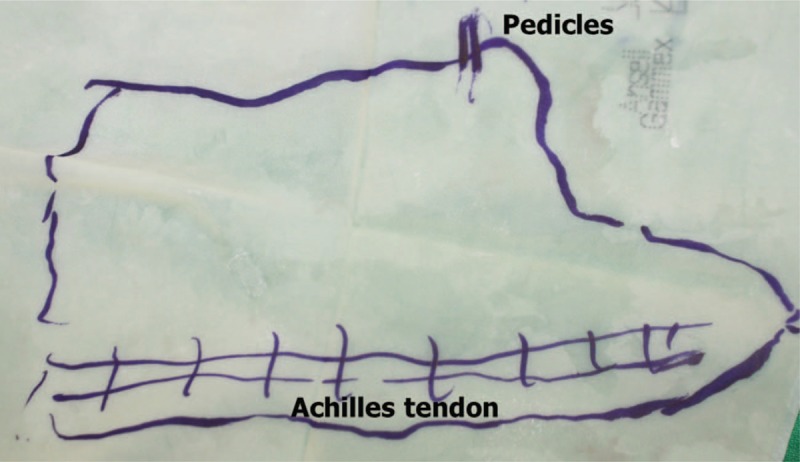
A template with surgical glove, which included the site of pedicles, Achilles tendon and contour of defect.

**Figure 4:**
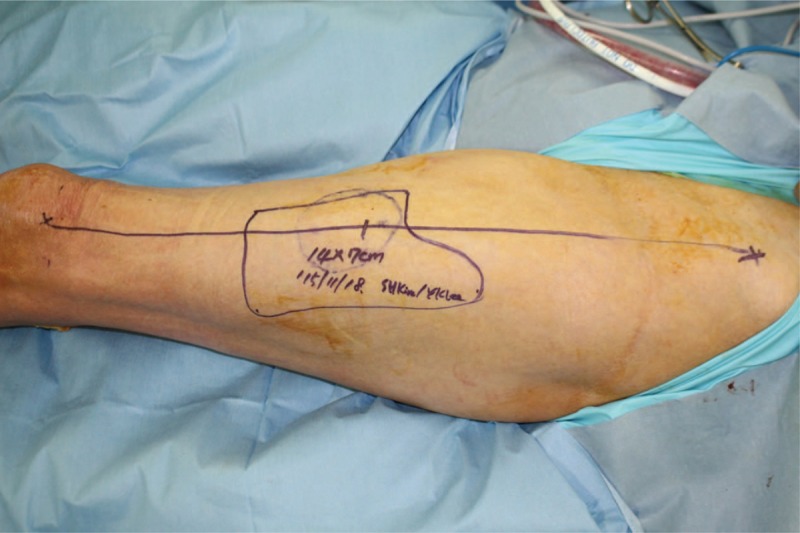
Intraoperative photograph showing the design of the composite anterolateral thigh flap including a skin paddle (14 × 7 cm) with vascularized fascia lata of the left thigh.

**Figure 5:**
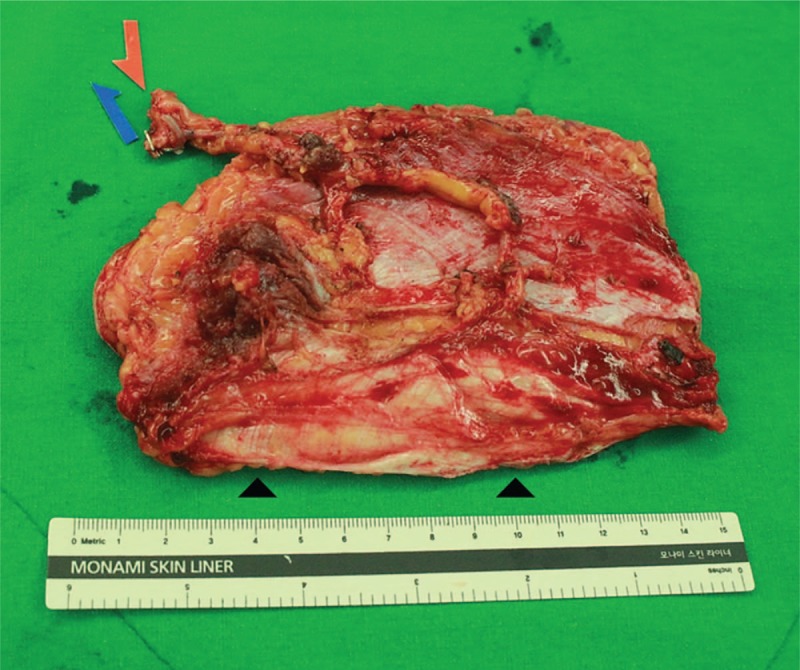
A 14 × 7 cm composite anterolateral thigh flap including a strip of fascia lata (arrowheads) at the left thigh.

**Figure 6:**
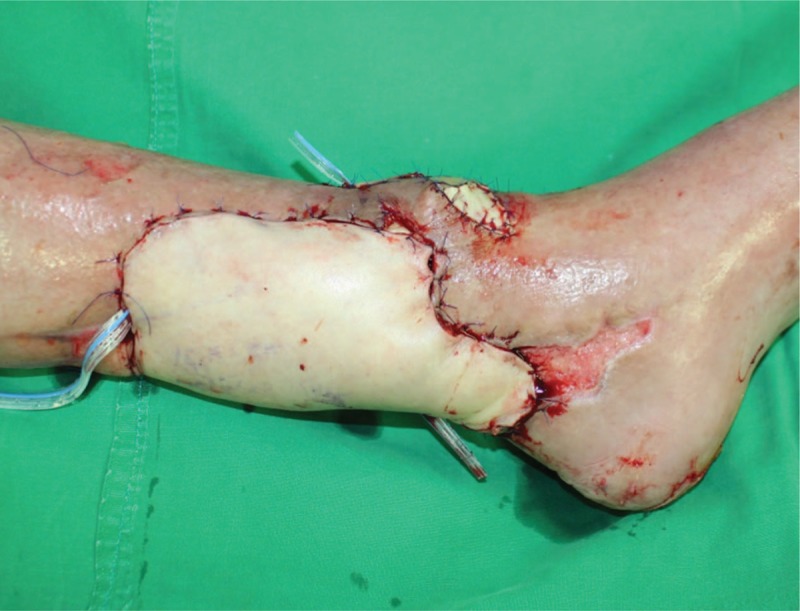
Immediate postoperative view.

We performed an operation with the patient placed in the supine position. All infection associated with necrotic Achilles tendon in the proximal muscle tissue was excised (Fig. 2). After debridement, the patient had a 16 cm tendon defect from the muscle with the ankle joint in neutral position. He had 2 cm of the distal tendon attached to the calcaneus. We extended the skin incision to the outside of the zone of injury in the anterior aspect of the ankle, dissected anterior tibial artery and vena comitantes to perform vascular anastomosis out of injury zone. We made a template with surgical glove, which included the vascularized fascia lata for the reconstruction of Achilles tendon (Fig. 3). We used the already manufactured template on the ipsilateral thigh, centering the flap over the perforator and drew the flap larger than the recipient site (Fig. 4). A 14 × 7 cm ALF flap with a large piece of fascia lata (bilaterally, approximately 2 cm extra fascia is taken) was harvested (Fig. 5). The donor defect was closed primarily over a silicon drain. For the reconstruction of tendon, the fascia lata was repaired first using multiple figure-eight sutures and modified Becker method^[8]^ with 4–0 prolene sutures at the separated end-to-end of the Achilles tendon. An end-to-end microvascular anastomosis was performed between the anterior tibial vessels and the flap pedicle vessels using 9–0 sutures microscopically after inserting the flap into the defect (Fig. 6).

Postoperatively, the ankle and leg were wrapped in a bulky dressing and immobilized with an above-knee splint and the flap was monitored intensively for 7 days. Anticoagulation therapy with prostaglandin E1 (10 μg/day) and heparin (5000 units/day) were administered for 1 week and aspirin 100 mg once a day for 4 weeks after the surgery. The flap survived completely without complications. Passive and active exercise of the ankle joint was started at 6 weeks after surgery. Subsequently, the patient underwent a graduated rehabilitation program, from a non-weight bearing exercise to partial-weight bearing exercise. Twelve weeks after the surgery, the patient was permitted full-weight bearing with gait training.

**Figure 7:**
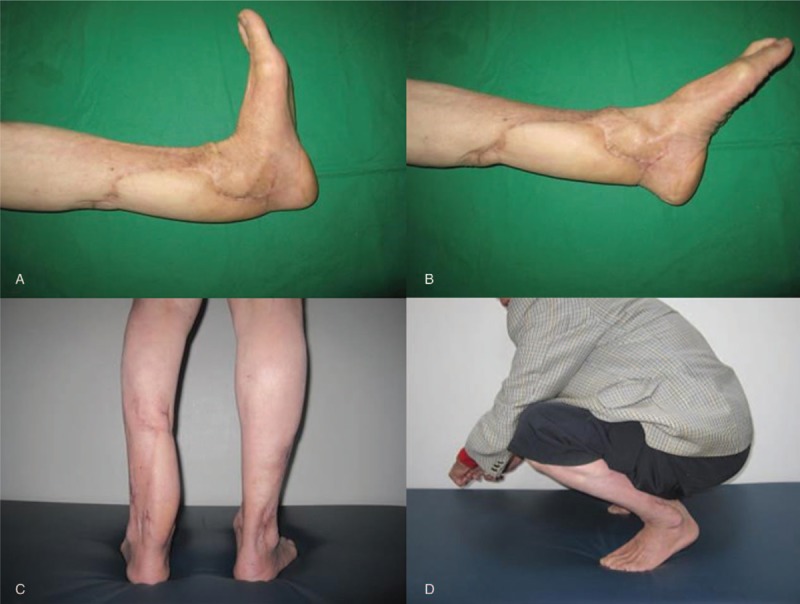
At 12 months, the follow-up photographs showing a 15° dorsiflexion (A), 45° plantar flexion (B), left heel with stable coverage (C), and the patient was able to squat (D).

At 12 months of follow-up, the patient was able to resume full daily activities, felt a little discomfort at the donor site after more than 2 h of hiking, but was able to walk without pain and without the need for support, also was able to squat, showed an ankle range of motion of 15° dorsiflexion and 45° plantar flexion, and the American Orthopaedic Foot and Ankle Society (AOFAS) score was 94 (Fig. 7).

## 4 Discussion

Treatment of an infected Achilles tendon is particularly difficult because of the poor vascularity of the tendon itself and the thin surrounding soft tissue. Therefore, the treatment principle includes meticulous debridement of the infected necrotic tissue and drainage of the abscess followed by intravenous antibiotic therapy. Reconstruction or reinforcement of the Achilles tendon is needed to treat the tendon defect created by debridement for restoration of functional plantar flexion.^[3]^ Skin graft or flap surgery is also often needed to cover the soft tissue defect.

Studies reported satisfactory results for the treatment of infected Achilles tendon without secondary tendon reconstruction, especially in an elderly patient.^[9–11]^ However, it is accepted that the Achilles tendon plays a critical role in normal ankle joint movement, maintenance of gait and in strenuous activities such as running, jumping or stair climbing. Although the patient described in our case is elderly, he was very active and used to hike daily for 2 h, before the injury. We, therefore, anticipated that Achilles tendon reconstruction would lead to better functional recovery of the patient, despite old age.

The free flap is the best option for the reconstruction of Achilles tendon defect after radical debridement, especially in the presence of an overlying soft tissue defect. Various types of free flaps have been proposed. Among these, the radial forearm flap with the palmaris longus tendon,^[12]^ lateral arm flaps with the triceps tendon,^[3]^ dorsalis pedis flap with the extensor digitorum longus tendon^[13]^ and the groin flap including the vascularized external oblique aponeurosis^[2]^ have been used as a composite-free tissue transplantation. The advantages include high resistance to infection, faster healing, fewer adhesions, and better gliding capability. However, these flaps have been used to small-sized defects. In our case, the soft tissue defect was 12 × 5 cm and the Achilles tendon defect was 16 cm. The defect size was relatively large to reconstruct using the flap mentioned above. Whereas the ALT flap is a very versatile flap, it has the advantage of large flap territory, durable skin quality, free from design, long vascular pedicle, and 3 to 4 mm thickness.^[7]^ Many authors have reported the use of composite-free ALT flap with the fascia lata for the reconstruction of composite Achilles tendon defects since the first description by Lee at al in 1999.^[4,7,14,15]^ The fascia lata is rolled up to serve as a vascularized tendon graft. However, in our case, we did not roll up the fascia lata, instead, it was sutured to the end of the expanded sole muscle, and at the distal point it wrapped the remnant of the Achilles tendon. We obtained a near full recovery of range of motion of ankle after reconstruction. Compared with the other fascia lata repair method, no large posterior expansion of the fascia lata was needed, which decreased donor site morbidity with functional reconstruction. Furthermore, the operation time also decreased with economic usage of fascia lata.

The limitation of the present study is that magnetic resonance imaging (MRI) evaluation was not possible at the final follow-up for Achilles tendon reconstruction due to patient refusal. It was confirmed that one-stage Achilles tendon reconstruction using a composite free ALT flap with the fascia lata provides good results, even in elderly patients. We expect that this method will be used for more cases in the future.

## 5 Conclusion

The treatment of infected Achilles tendinitis with overlying soft tissue defect still remains a challenging issue. The free ALT composite flap with vascularized fascia lata was successfully used for the treatment and provided good functional and cosmetic outcomes even in an elderly patient.

## Author contributions

**Conceptualization:** malrey lee.

**Writing – original draft:** Young Keun Lee, malrey lee.

**Writing – review & editing:** Young Keun Lee, malrey lee.
